# Individual variability in foraging success of a marine predator informs predator management

**DOI:** 10.1038/s41598-022-15200-y

**Published:** 2022-07-01

**Authors:** Grace Freeman, Erin Matthews, Erin Stehr, Alejandro Acevedo-Gutiérrez

**Affiliations:** 1 Western Washington University, Bellingham, WA USA; 2 Skagit Fisheries Enhancement Group, Mount Vernon, WA USA; 3 Present Address: WI Department of Natural Resources, Office of Applied Science, 2801 Progress Rd, Madison, WI 53716 USA

**Keywords:** Behavioural ecology, Conservation biology, Ecosystem services

## Abstract

The complexities of trophic dynamics complicate the management of predator populations. Targeted culling campaigns are one management strategy meant to control predation for the benefit of the prey population. In these campaigns, individual predators are often considered “rogue” based on visitation rates to the site of concern. This definition assumes that all predators impact prey equally. However, individual variability in foraging success may compromise this assumption. To examine this hypothesis, we studied harbor seals preying on adult salmonids during the 2014–2019 fall runs in Whatcom Creek, Bellingham, Washington, USA, and recorded visitation rate and foraging success of individual seals from photographs and field observations. We then used Generalized Linear Mixed-Effects Models to model individual foraging success. Models including harbor seal identity better explained foraging success than models based on visitation rate alone. We concluded that considering intraspecific variability and classifying “rogue individuals” based on foraging success is a more accurate protocol for managing predator populations than relying solely on visitation rate of the predators.

## Introduction

Predators often affect their communities in ways disproportionate to their biomass. This is especially the case for keystone species but holds true for meso-predators as well due to cascading effects of predation through a trophic web system^1,2^. Consequently, resource managers targeting the protection of a prey species may focus management actions on the predators. General culling and bounty campaigns in which predators are killed or hunted at large have long been employed to control predator populations in both terrestrial and marine settings^3,4^. These general campaigns target a predator population as a whole with the goal of decreasing predator abundance and as a means of reducing prey mortality^5^. Such management approaches have historically been based on the assumption that predator populations consist entirely of generalists; that is, individuals that forage opportunistically rather than seeking out one type of prey or specializing in a given foraging behavior^6^. Recent, targeted culling campaigns remove individual predators thought to have the largest impact on prey species of concern^5^. The targeted individuals are known as “rogue individuals” and are thought to consume a disproportionately large amount of prey relative to others in the same population^7–9^. An underlying assumption in the rogue individual paradigm is that a small number of individuals in a predator population are responsible for most of the depletion of the prey population^9^. Under this assumption, directed culling campaigns have been used in which presumed rogue individual predators are targeted for removal to reduce prey mortality while simultaneously maintaining stability of the predator population^5,10^. Targeting rogue individuals addresses the flawed assumption that all predators impact the prey population to an equal degree, however, there is a dearth of research and scientific data for use in culling campaigns, especially in marine environments^5,11^.

Intraspecific variation in prey consumption and resource use by predators can significantly influence prey community structure across a variety of predator taxa^12,13^. For example, in California sea lions (*Zalophus californianus*), males tend to forage on large pelagic species such as adult salmonids (*Oncorhynchus* spp.) and females target juvenile fish and benthic prey^14,15^. In sea birds and polar bears (*Ursus maritimus*), males forage at different times of day from their female counterparts and typically consume prey at a higher trophic level^16,17^. There are even reports of male grey seals (*Halichoerus grypus)* that specialize in raiding human-placed salmon traps^18^. Moreover, individuals can differ in how well they perform adaptive behavior for reasons not attributed to sex or visitation such as variances in either physical or behavioral skill^19^. For example, individual foraging variability in sea otters (*Enhydra lutris*) and harbor seals (*Phoca vitulina*) has been attributed to differences in dive behavior^20–23^. Alternatively, male southern sea lions (*Otaria byronia*) seem to display prey preferences given that most rarely consume fur seals (*Arctocephalus australis*), but others have a tendency to do so repeatedly^24,25^. Accounting for variability among individuals of the same species—whether attributed to sex or individual differences—is an essential but often overlooked step in effectively managing predator populations.

The success of targeted campaigns hinges on the ability to identify which individual predators have the largest impact. Most current management strategies assume a homogeneous predator population or account for individual variability based solely on the amount of times a predator visits a site of interest^5,9^. In the United States, permits for targeted campaigns against marine mammals like pinnipeds, are granted based on repeated appearances of individual predators at the site of concern^26^. For instance, in the culling campaign at Bonneville Dam in Oregon, USA, an individual is considered rogue and eligible for removal if it is observed at the site on five or more days (regardless of timing) or is observed preying on a species of concern at least once^27^. Using visitation rate alone to define rogue status assumes that all predators impact the prey population equally and does not address individual variability in foraging success. Yet, intraspecific variation in prey consumption by predators significantly impacts prey communities^12,13,19^. As such, accounting for intraspecific variability among individuals is crucial to effectively manage predators via culling.

Harbor seal predation on salmonids in the Salish Sea (the inland waters of Washington State, USA, and British Columbia, Canada) is an ideal study system to examine the assumption that visitation rate is the sole driver of variability in the consumption of prey. Harbor seal populations on the west coast of the United States have been growing at an estimated rate of 5–7% annually since the 1970s^28^ a recovery which has shifted trophic dynamics and dramatically increased predation pressures on salmonids of conservation concern^14,29–31^. Moreover, they aggregate at river mouths to prey on returning salmonids. One such site is Whatcom Creek in downtown Bellingham, Washington State, USA, which borders a public park and hosts a salmon hatchery^32^. This unique combination offers the opportunity to estimate salmonid abundance, identify individual harbor seals, and record harbor seal occurrence and foraging success. Here, we show that individual variability in foraging success prevents the use of visitation rate alone as a proxy to classify an individual as rogue. This finding contradicts the assumptions currently being used to inform predator management strategies and suggest a more precise approach.

## Results

**Figure 1:**
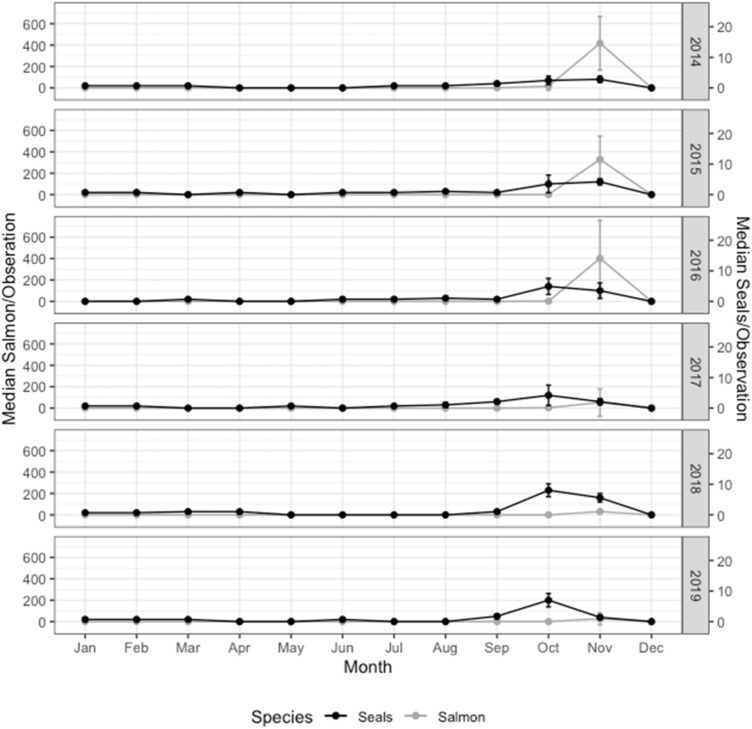
Median number of harbor seals present at each observation (right y-axis) and median number of salmonids present per observation relative to month and year (left y-axis). Salmonid medians were calculated as the median of a three-day rolling average corresponding to the day of observation. Error bars represent interquartile range to show spread of the data.

We identified 170 individual harbor seals, of which the majority (91.7%) were recorded only during salmonid run months (Supplementary Fig. S1). Over half of the seals (56.5%) were observed during more than one year, seven (4.1%) individuals were observed during five of the years, and only one (< 1%) individual was observed at the creek during all six years of the study. There was no evidence of individuals foraging outside of the run. The mean salmonid run size per year was 6494 individuals (± SD 6302; n = 6 years) with a minimum of 191 and a maximum of 14,611 salmonid returns per year. The run started in late October, peaked in November, and declined in December (Fig. 1). Given that 96% of the returns were hatchery-raised Chum salmon (*O. keta*), we combined all salmonids species into one metric. During years with large salmon runs (2014–2016), harbor seal occurrence followed the same pattern as the salmon abundance with a peak in November (Fig. 1). In years with relatively small runs (2017–2019), seal numbers continued to peak in the fall despite a much lower peak in salmon abundance at that time (Fig. 1). Visitation rate of individual seals varied across the study: the median number of visits by an individual seal per run was 1.33 (IQR = 1.77; n = 170 seals) with a minimum of 0 and a maximum of 10.5 visits per run.

**Figure 2:**
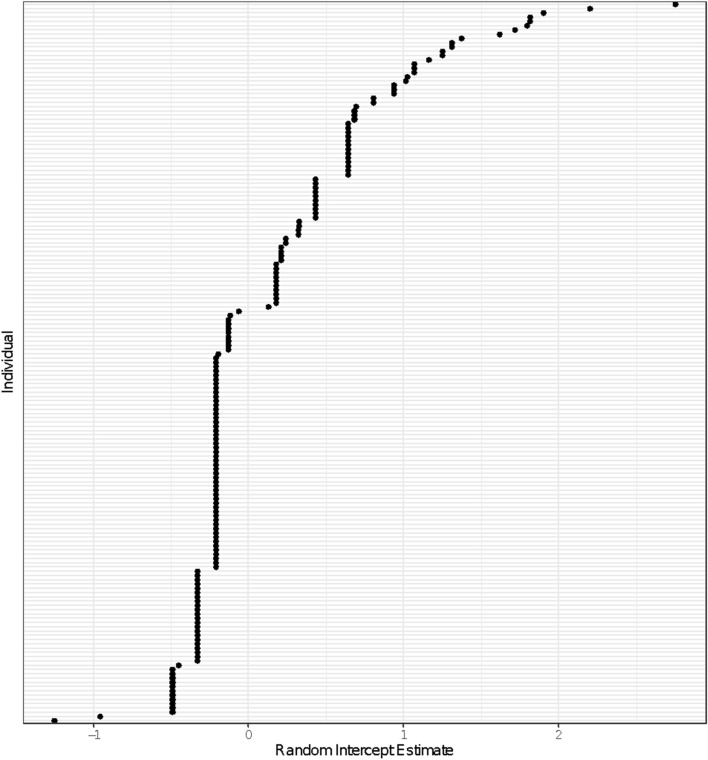
Estimate of random intercept for each individual seal (n = 170) based on the full model of best fit. The variability in random intercepts for each individual seal illustrates the importance of including ID as a random factor in the model.

**Figure 3:**
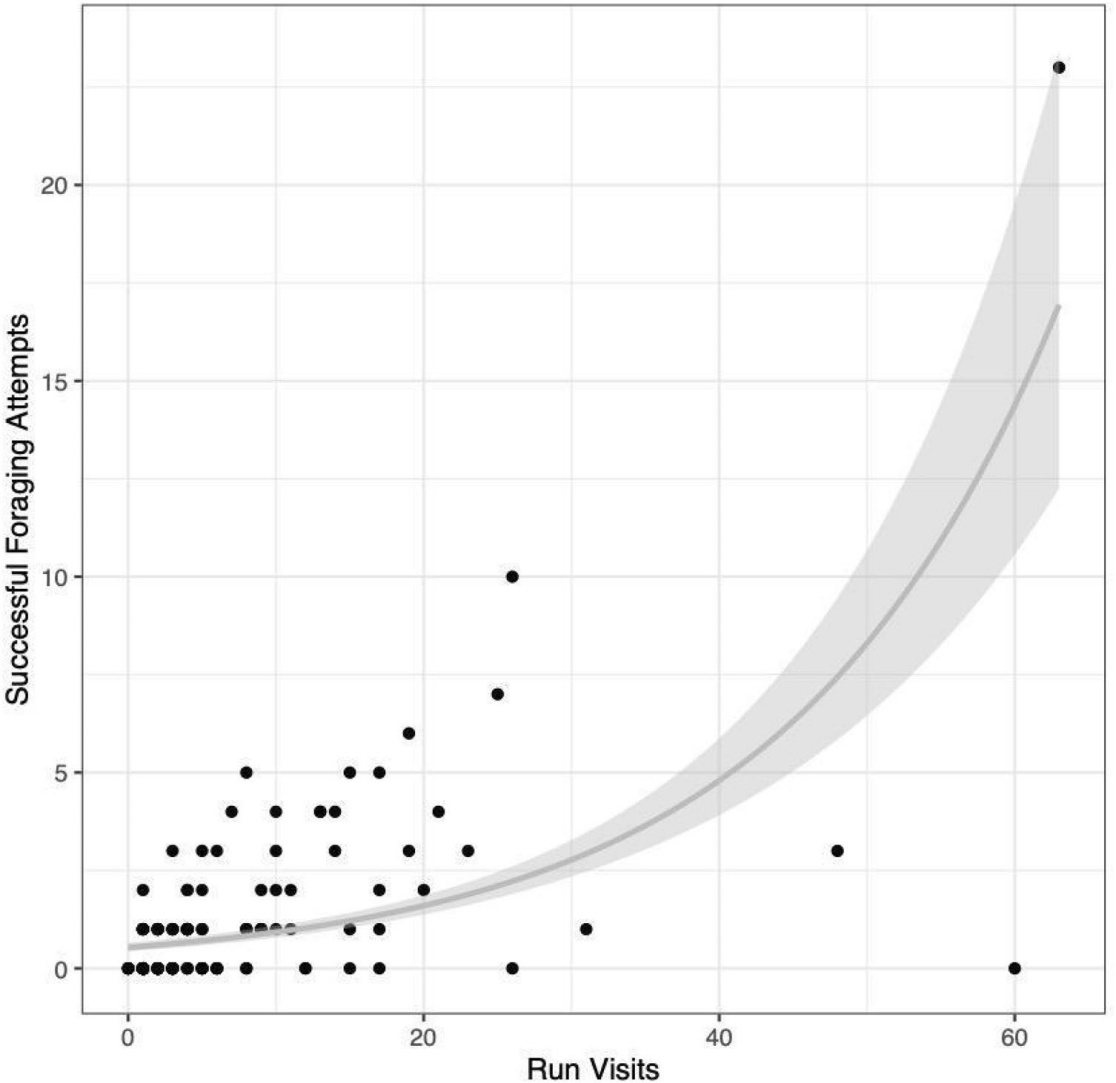
Total successful foraging events relative to run visits for each individual harbor seal (n = 170). The line represents a Generalized Linear Model (GLM) of successes by run visits with a Poisson distribution (adjusted R^2^ = 0.36) and 95% confidence interval based on standard error.

Total foraging success varied among individual harbor seals during the run. We observed 164 successful attempts, with 66 (38.8%) individual seals having one or more foraging successes (Supplemental Fig. S2). The total number of foraging successes per individual ranged from 0 to 23 with a median of 0 and an IQR of 1. Mean salmonid abundance (the sum of salmonid abundances during each visit calculated as a three-day rolling average divided by the number of visits for each individual) was a significant predictor of the total number of successful foraging attempts (Table 1A). Run visits, total visits, and seal ID were all significant in their respective null models (Table 1A). The null model with seal ID added as a random intercept provided the best fit, with an AIC significantly lower than that of the run visits model (ΔAIC = 14.0; Table 1A; Fig. 2) and explained significantly more variance than the model based on visits alone (adjusted R^2^ = 0.81 and 0.36 respectively; Fig. 3). Of the 11 models tested, the best full model included seal ID as a random intercept and the number of years during which an individual was observed during the run (run years) as a fixed factor (Table 1B).

**Table 1:** (A) Model results predicting the total number of successful foraging attempts recorded by individual harbor seals relative to number of run visits, number of total visits, and individual ID. The change in AIC value is the difference between the tested model and the model of best fit (as determined by the lowest AIC value). (B) Generalized Linear Mixed-Effects Model (GLMM) output for the final, most parsimonious model describing the total number of successful attempts recorded by an individual harbor seal.

**(A)**					
Models for total successes		Estimation method	df	AIC	ΔAIC
Successes ~ Mean Salmon		GLM	2	606.0	204.1
Successes ~ Mean Salmon + Total Visits		GLM	3	502.8	101.0
Successes ~ Mean Salmon + Run Visits		GLM	3	452.9	51.0
Successes ~ Mean Salmon + (1|ID)		GLMM	3	438.9	36.9
Successes ~ Run Years + (1|ID)		GLMM	3	401.9	0

**(B)**					
Random effect		Variance		N	
ID		1.14		169	

Fixed effect	Estimate	95% CI	SE	Z value	*P* value
Run years	0.57	(0.39, 0.74)	0.08	6.34	<< .001

CIs represent the 95% confidence interval for the estimate of each parameter in the model. Note that a *P* value cannot be reliably calculated for random effects in mixed models and thus has been omitted from the table.

**Figure 4:**
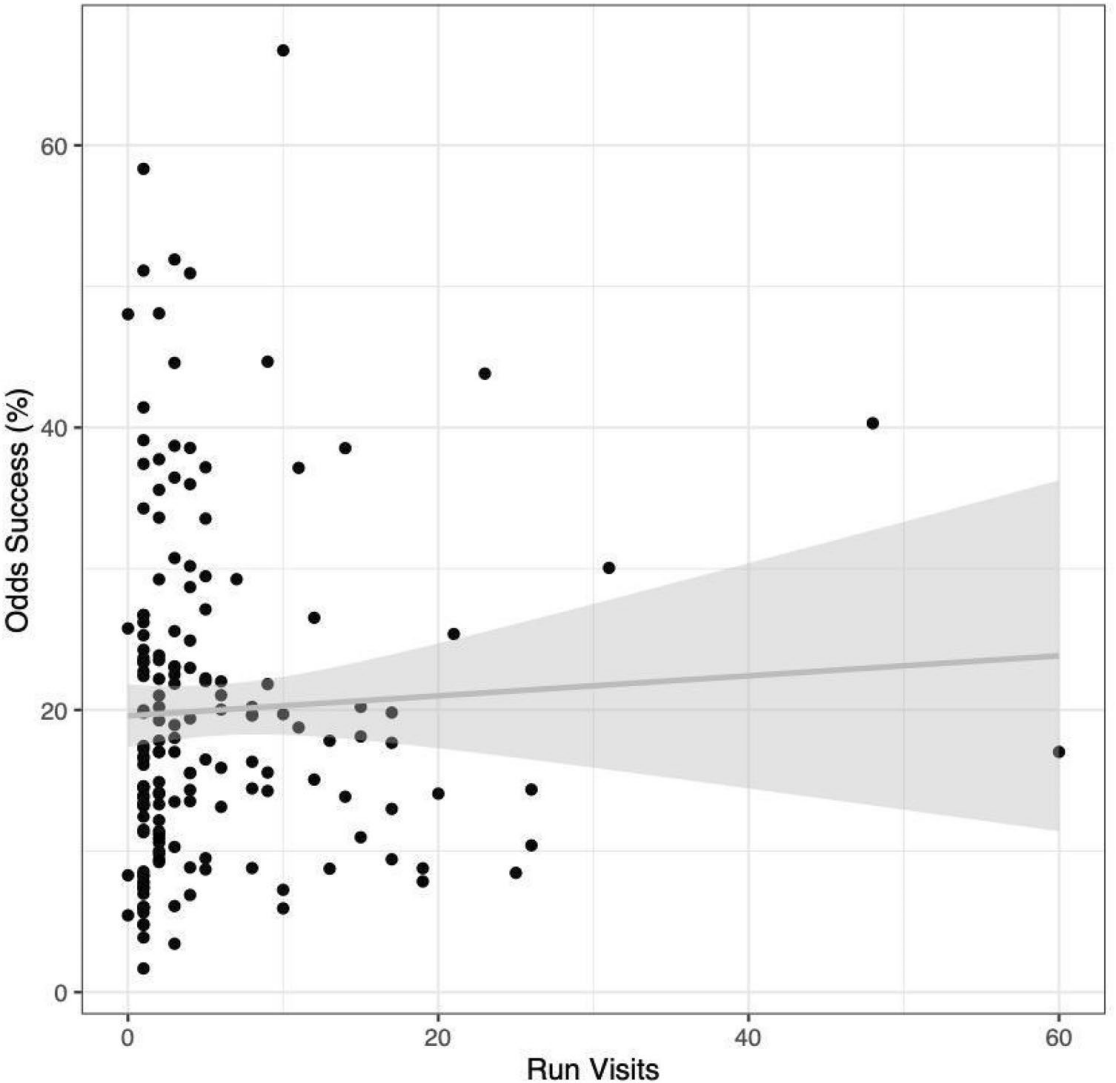
There is no relationship between the odds of a successful foraging attempt for each individual harbor seal (n = 170 seals) relative to run visits. The line represents a GLM of predicted odds of success by run visits with a binomial distribution based on a visits-only model (R^2^ = 0.002) with a 95% confidence interval based on standard error.

The odds of a successful foraging attempt for each individual seal also varied greatly. There was no relationship between the number of run visits and odds of success on any given attempt (R^2^ = 0.002; Fig. 4).

Salmonid abundance varied across the study and was a significant predictor of odds of success during a given observation period (Table 2A). Neither the total number of site visits, nor the number of run visits was significant in their respective null models (Table 2A). The inclusion of seal ID into the null model based on salmonid presence was significant and improved the candidate model fit (ΔAIC = 37.7; Table 2A). The best full model of the six models tested included the number of fishermen present, the number of seals present, the interaction between these two as fixed factors, and seal ID as a random intercept (Table 2B).

**Table 2:** (A) Model results predicting the odds of success of a single foraging attempt by an individual harbor seal relative to number visits for that seal as well as identity of the seal in question. The change in AIC is the difference between the tested model and the model of best fit (as determined by the lowest AIC value). (B) GLMM model output for the final, most parsimonious model describing odds of success for a given foraging event.

**(A)**					
Models for odds success	Estimation method	df	AIC	ΔAIC	
Odds Success ~ Salmon	GLM	2	915.2	70.1	
Odds Success ~ Salmon + Total Visits	GLM	3	914.3	69.2	
Odds Success ~ Salmon + Run Visits	GLM	3	916.7	71.6	
Odds Success ~ Salmon + (1|ID)	GLMM	3	877.4	32.3	
Odds Success ~ Fishermen + Other Seals + Fishermen: Other Seals + (1|ID)	GLMM	5	845.1	0	

**(B)**					
Random effect		Variance		N	
ID		0.69		1111	

Fixed effects	Estimate	95% CI	SE	Z value	*P* value
Fishermen	0.061	(0.041, 0.081)	0.01	5.98	<< .001
Other Seals	0.076	(0.042, 0.110)	0.02	4.37	<< .001
Fishermen:Other Seals	− 0.002	(− 0.004, − .001)	< 6.25E−4	− 3.88	<< .001

CIs represent the 95% confidence interval for the estimate of each parameter in the model. Note that a *P* value cannot be reliably calculated for random effects in mixed models and thus has been omitted from the table.

## Discussion

Individual harbor seals exhibited variable foraging success, whether measured cumulatively or by odds of a successful attempt. Even though total foraging success and number of run visits were correlated, there was significant variance left unexplained by a visits-only metric (adjusted R^2^ = 0.36). Incorporating individual identity into the model explained most of the variance in total successful foraging attempts (adjusted R^2^ = 0.81). The notable increase in variance explained underscored the importance of considering individual identity when modeling cumulative success. Further, there was no relationship between odds of a successful foraging attempt and the number of run visits recorded by each individual (R^2^ = 0.002). Thus, we proposed that, instead of the number of site visits, predator management should focus on the foraging success of each individual predator.

There are several possible explanations for the observed individual variability in foraging success. The significance of “run year” in explaining an individual’s total foraging successes provided evidence for the importance of an individual’s site-specific experience. With each additional run during which a seal was observed, the predicted number of successful foraging attempts recorded by that individual also increased (Table 2A). This increase was independent of run visits, suggesting a potential behavioral or experience-based learning effect given that the individuals who return year after year are more successful hunters overall regardless of how many times they visit during those years. The significance of run year also corroborated the idea of ”habituated individuals” proposed by NMFS in the Bonneville Dam campaign, USA^26,33,34^. Previous work on marine^10,18^ and terrestrial^9^ predators suggested that those individuals who return to a site year after year are habituated and have a greater impact on the local prey population than those individuals who visit during only one season. Evidence from this study supported the hypotheses that repeat visitors across years are more impactful to the prey population than their non-habituated counterparts. This evidence was nuanced, however, in that those individuals who visited more often in one year were not more likely to record a success during each visit (Fig. 4). Further, the impact of run years was relatively small and not independent of individual predator identity (Table 2B). Hence, predator identity must still be considered in that it separated habituated individuals with high rates of success from individuals who visited the site frequently but have low predation success.

There were additional factors that may explain the individual foraging variability we observed, however, we were unable to measure them in this study. For example, sex played a role in harbor seal prey-preference^21,23^, but we were unable to measure sex in this study. Given that only one type of prey was considered, the differences in foraging success during the salmon run could have been attributed to sex of the seal. Skill could have also played a role in determining a predator’s impact on the prey population. Furthermore, size of the seal was impossible to measure in this study but would have been correlated with both sex and age (possibly translating to skill)^23^. The most successful individual (ID0039) was consistently observed hunting in the same location within the creek: at the base of the falls upstream from the hatchery’s fish ladder. Few other seals ventured high into the falls where ID0039 recorded most of its successes, leading us to propose that ID0039 may have possessed the size and skill required to swim against the rapids and falls. Finally, differences in foraging technique employed by each individual were another possible driver of individual predator variability. Specifically, some individuals showed preference for hunting relatively high upstream in the falls where salmon sometimes rest on their migration. Other individual seals seemed to prefer a more passive method of hunting termed “bank” in which the individual waited in the shallow eddy near the hatchery fish ladder and opportunistically cornered fish onto the bank. Another group of individuals seemed to prefer swimming upside down passively floating in the mid-channel and waiting to attack fish passing by them.

In addition to individual variability, other factors also influenced the odds of foraging success. The number of human anglers and the number of additional seals present during a foraging attempt increased the individual odds of foraging success (Table 2B). With each additional angler present, the individual odds of a successful foraging attempt increased by 6%. Observational evidence suggested that individual seals would target fish already on the anglers’ lines. Further, with each additional seal present, the individual odds of a successful foraging attempt increased by 8%. Although the reasons for this increase were unknown, observations of individual seals cornering a fish on the bank of the creek or against rocks to then share the catch led us to believe the use of cooperative hunting techniques was among them. Cooperative hunting has been documented in pinnipeds, such as adult leopard seals (*Hydrurga leptonyx*) luring penguins (*Pygoscelis antarctica*) toward another seal waiting below the ice^35,36^ or adult male Galapagos Sea Lions (*Zalophus wollebaeki*) working together to corner yellowfin tuna (*Thunnus albacares*) onto a bank before sharing the prey item(s) between them^37^. Yet, we could not discount additional potential explanations, such as prey sharing, scavenging, or simple opportunistic advantage. Regardless, the impact of the number of anglers and other seals present was independent of visits recorded by each predator, underscoring again the importance of individual variability beyond visitation rate alone.

Using Whatcom Creek as a case study, we evaluated current visitation-based protocols under which five visits or a single successful foraging attempt classifies an individual as rogue^28,33^; and compared these to our proposed protocols. Under our proposed criteria, a rogue individual was one with a number of successful foraging attempts greater than or equal to one standard deviation above the group mean. The mean was chosen for this threshold in lieu of median to account for the skew of successful foraging attempts made by individuals and explicitly select those with unusually high success. If the median and IQR were to be used, each system would be guaranteed a certain number of rogue individuals depending on sample size. With the use of mean and standard deviation, however, a population in which the number of successful foraging attempts by each individual fell within one standard deviation of the mean would have no rogue individuals, accurately capturing the lack of an individual with a disproportionate impact on the prey population. At Whatcom Creek, the mean number of successful foraging attempts per individual was 1.00 (± SD 2.28). Hence, a rogue individual was one with four or more successful foraging events. Current visitation-based protocols would have classified 81 (47.6%) rogue individual seals at Whatcom Creek and identify them for removal. However, 17 (21.0%) of those individuals never recorded a successful foraging attempt and 36 (44.4%) recorded only one. Our suggested protocol classified only 14 (8.2%) rogue individuals and removing them from the studied population would have eliminated more than half (51.5%) of the successful foraging attempts observed in the study. This discrepancy illustrated the outsized impact of the most prolific individual predators on their prey regardless of visit frequency and the importance of future study into individual variability among predators.

Our findings showed that the consumption of salmonids varied to a large extent with harbor seal individual identity, and there may have been even greater variability than we were able to observe. Some individuals regularly captured and consumed more than one salmonid during a given foraging event, but we were unable to measure the exact number in this study. Rather, a binary variable was measured to determine whether a seal had been successful on a given day, and this distillation, though necessary, limited conclusions. Furthermore, a protocol was stablished to determine if a photo of a seal with a salmon represented a true capture or a scavenging event. Though unlikely, it was possible for some scavenging events to be tallied as captures and vice versa. As a result, examining the absolute number of captures recorded by each individual—rather than the number of successful foraging—would have provided a clearer measure of each predator’s impact. Additionally, a rolling average was used in this study as a proxy for the number of prey individuals present at the site on a given day. Describing this value at a finer scale would have helped parse the effects of prey abundance on the success of predators.

Managing predator populations for the benefit of their prey, relies on the assumption that predation is the main limiting factor, or one of the most significant limiting factors, affecting a declining or at-risk stock. Yet successful prey management must examine other limiting factors; in the case of salmonids, these include pollution, climate change, habitat loss, and overharvesting by humans^38,39^. Including other factors will contextualize the relationship between predator and prey within the given system and allow managers to make informed decisions on whether to pursue predator removal^5,10^.

Predator removal actions, like culling, have further relied on the assumptions that all individuals within the predator population are equally likely to impact the prey population and that those individual predators who visit more frequently will consume more prey^9^. This study presents evidence of individual variability in predator foraging success that challenges these assumptions. Top resource limiting factors are often anthropogenic in source^40^, but if predator management must be pursued, modifying the criteria by which rogue individuals are defined could inform better management strategies across environments and predator–prey systems both terrestrial and marine. This study finds that a new definition of rogue or problem individual based on foraging success would be a more effective management tool.

We are aware that it is more challenging to describe the foraging success of an individual seal than its visitation rate to a particular site. However, many conflict sites where seals aggregate to prey on adult salmonids are choke points where foraging observations can be conducted, such as Bonneville Dam in Oregon, USA^10^ or near finfish farms such as those common in Scotland^41^. Furthermore, because individual predators must be identified to determine visitation rate, tracking successful foraging attempts would require only a small shift or increase in sampling effort. In this study, we relied on the effort of undergraduate student volunteers to conduct the observations and take photographs of seals. We imagine that local volunteers could accomplish this effort at other locations given the establishment and increase in the involvement of the local community and citizen scientists in the scientific endeavor^42^. Consequently, we propose that it is feasible to establish a management approach that determines problem individual pinnipeds based on foraging success and visitation rate rather than on visitation rate alone.

## Methods

The Whatcom Creek study area (48° 45′ 14″ N and 122° 29′ 00″ W) is approximately 215 m long, 25–58 m across, and covers a surface area of ca. 7225 m^2^. Harbor seals are safely observable from a path to the southeast of the creek and from a boardwalk built along the north bank of the creek. The creek supports small wild runs of Coho (*O. kisutch*) and Steelhead (*O. mykiss*)^43^, and the Whatcom Creek Hatchery maintains a population of chum salmon^44^. We used six years of data collected at Whatcom Creek from 2014 to 2019.

All data collection complied with the Marine Mammal Protection Act^26^ and did not require additional permitting as determined by Western Washington University’s Animal Care and Use Committee. Undergraduate students from Western Washington University collected data on harbor seal occurrence at the creek for two-hour observation periods during daylight slack tides. At the start of each observation and every 30 min for the remainder of the time, observers also recorded the number of human anglers. Harbor seal behavior was observed for the entirety of the observation period. Any seal occurrences during October-December were tallied as “run visits”.

Given that the site was small and harbor seals were typically alone or in pairs, observers were able to employ focal individual sampling^45^ on every seal. We classified behavioral states based on behavioral events at the surface. “Socializing” meant that two or more seals were within two body lengths of one another and visibly interacting (e.g., fighting over prey, vocalizing toward each other). “Hunting” referred to any seal that was floating and/or actively swimming and engaged in quick bursts of speed as if pursuing prey, confirmed on many occasions from direct observations of salmon swimming ahead of the seals. During the latter years of the study, we further divided this behavioral state into four foraging techniques. “Wake” meant a seal was pursuing prey under the water in a manner that produced a wake on the surface. ” Bank” referred to one or more seals cornering a salmon onto the bank before lunging at it. “Upside down” was used when a seal was floating upside down at the surface of the water and moving its head sideways, as if scanning for prey. Finally, “Parked” referred to a seal positioned near human fishers and lunging directly at their angling lines. “Eating” was a behavioral state selected when a seal had control over a fish and was consuming it at the surface. In such a case, observers attempted to determine whether the prey had been captured by the eating individual or scavenged/stolen from another seal. Seals were classified as successful only when they were observed eating, which was possible because pinnipeds capture large prey under the water and quickly bring it to the surface to consume^35,37^. For analysis, the above behavioral states were distilled to a metric of eating or not eating which was later confirmed a posteriori via photo analysis.

To identify individual seals, observers used a digital camera with a 75–300 mm, f/4–5.6 lens to take right, left, and front head photos every time a seal surfaced. These photos were entered into a database and run through the software Wild.ID^46^ to propose possible matches which were then verified. If Wild.ID failed to find a match for a new photo, the photo was identified manually or assigned a new ID number. We employed standard methodology^47,48^ to identify individuals by comparing distinctive spot patterns, scars, eye color, and other features to those present in the database of all individuals observed at the creek. For a manual match to be confirmed, at least three features on the unknown individual had to match exactly with a database photo and a second experienced researcher had to confirm the match to address potential observer bias. Sex and size of the individual seal could not be determined.

To estimate salmonid abundance, we used Whatcom Creek Hatchery’s record of daily returns^49^. Because hatchery staff did not collect return numbers in real time, but summarized them at the end of the day, we calculated a 3-day rolling average of salmonid numbers the day before, during, and after an observation. A mean was used for this calculation to temporally smooth the variable. We then used this value as an indicator of total salmonid abundance at the time a seal was observed.

Since prior research^32^ and field observations indicate that harbor seals primarily visit the site during the salmon run, we assumed that they were present at the creek during such times to hunt. Thus, we tallied each seal visit during the run as a foraging attempt. Every surfacing event was photographed, and successes were confirmed a posteriori via photo analysis by determining the presence or absence of adult salmonid in the seal’s possession. To address the possibility that a prey item was stolen or part of it scavenged from another seal, an individual was not considered successful unless pictured with an intact or nearly intact salmon. If a seal was seen with a small scrap of fish (without having previously been seen with the whole fish), it was assumed to be a scavenging event rather than a successful foraging attempt. This distinction was possible because pinnipeds capture their larger prey under the water and quickly bring it to the surface to consume^35,37^. Thus, it was possible to document all eating behavioral states that occurred during observations, and each successful foraging attempt was assumed to be associated with a photo of the successful captor in possession of an intact or nearly intact fish. We could not quantify the exact number of prey items consumed by an individual during each successful foraging attempt. Thus, we defined successful foraging attempts in a binary fashion: as those attempts in which a seal captured one or more salmonids during the two-hour observation period.

We conducted all analyses with R statistical software version 4.0.2^50^. We ran two analyses to describe individual harbor seal foraging success. The first analysis examined the total number of successful foraging attempts made by an individual over the six years of the study. Current management practices are more concerned with an individual predator’s effect on the prey population (total successes) rather than its success rate. Hence, we used the number of successful foraging attempts as a proxy to represent a seal’s overall success. For this analysis, we used Generalized Linear Mixed-Effects Models (GLMM) with a Poisson log link function for models including a random factor. Generalized Linear Models (GLMs) were used for fixed-effect only models designed to mimic techniques currently used in management in which individual ID is ignored and all observations are treated as independent. The set of models collated each individual’s foraging attempts, thus describing its collective impact over the years. The second analysis examined the odds of foraging success for each attempt. For this analysis we used a GLMM with binomial distribution in the R package “lme4” to account for the binary nature of the independent variable^51,52^. This set of models considered each individual foraging attempt, thus allowing the inclusion of variables that could influence the success of each attempt. For both sets of models, we calculated adjusted R^2^ values with the “rsq” package^53^.

We validated model assumptions with residual plots and assessed multicollinearity among variables with a Variance Inflation Factor (VIF) calculated with the “performance” package^54^. If a covariate was collinear with another in the model (VIF > 10), the factor with the highest VIF value (thus explaining the most overlapping variance) was removed and the model was run again. Once multicollinearity was addressed, we used the lowest Akaike Information Criteria (AIC) value to indicate the most parsimonious model^55,56^.

Each set of analyses (one for total number of successful foraging attempts and one for odds of a successful foraging attempt) consisted of two steps: The first step involved building null models based on seal ID and the visits metrics (run and total). Though only run visits were considered foraging attempts, “total visits” was included in a candidate model in an effort to mimic current practices used to determine rogue status which examine predator presence at times outside of runs or seasons of high prey abundance^26,27^. In addition, visits to the site outside of the run may also increase future successful foraging events by familiarizing a seal with the site. Thus, we tested the effects of both “run visits” and “total visits” in the first step of model evaluation. In the second step, we determined the best full model using a backwards-directional model selection technique with potential factors and their interactions added based on ecological knowledge of the system and tested at each step^56^. When fitting the full model for the total number of successful foraging attempts made by a given individual harbor seal, the following factors and their interactions were considered in candidate models: mean number of fishermen present during an observation was included as a fixed factor because field observations of the “parked” hunting technique suggested that the presence of human fishermen at the site could increase seal successes via opportunistic foraging. A 3-day rolling average of salmon present at the site was included as a fixed factor to address the assumption that an increase in salmon present at the site during an observation would lead to an increase in successful foraging attempts. “Run years” was tested as a fixed factor to account for a potential learning effect derived from repeated years of hunting at the same site. Finally, seal ID was included in the candidate model as a random factor to address the repeated sampling that occurred at the site and account for intrinsic variability among individual seals.

To determine the full model explaining the odds of a successful foraging attempt, candidate GLMMs again included number of fishermen and number of salmon present at the time of observation as fixed factors. The shallow nature of the study site meant that at low tide, a significant amount of rock was exposed and movement through the shallow water became more difficult for the seals. Thus, the odds model candidates also included tide level (high or low) as a fixed, categorical factor. The number of other seals present at the time of the observation was included as a fixed factor based on behavioral observations pointing to the use of cooperative hunting and the potential increase in success rate associated therein. Finally, seal ID was again included as a random factor to address individual variability among seals.

### Ethics statement

All data collection complied with the Marine Mammal Protection Act^26^ and did not require additional permitting as determined by Western Washington University’s Animal Care and Use Committee.

## Supplementary Information

Supplementary Figures.

## Data Availability

Supporting data are available upon request from Dr. Acevedo-Gutiérrez.

## References

[1] Krause M, Robins K (2017). Charismatic species and beyond: How cultural schemas and organisational routines shape conservation. Conserv. Soc..

[2] Marshall KN, Stier AC, Samhouri JF, Kelly RP, Ward EJ (2016). Conservation challenges of predator recovery. Conserv. Lett..

[3] Bearzi G, Holcer D, Di Sciara GN (2004). The role of historical dolphin takes and habitat degradation in shaping the present status of northern Adriatic cetaceans. Aquat. Conserv. Mar. Freshw. Ecosyst..

[4] Lavigne DM, Gales N, Hindell M, Kirkwood R (2003). Marine mammals and fisheries: The role of science in the culling debate. Marine Mammals: Fisheries Tourism and Management Issues.

[5] Bowen WD, Lidgard D (2013). Marine mammal culling programs: Review of effects on predator and prey populations. Mamm. Rev..

[6] Svanbäck R, Persson L (2004). Individual diet specialization, niche width and population dynamics: Implications for trophic polymorphisms. J. Anim. Ecol..

[7] Butler JRA (2008). The Moray Firth Seal Management Plan: An adaptive framework for balancing the conservation of seals, salmon, fisheries and wildlife tourism in the UK. Aquat. Conserv. Mar. Freshw. Ecosyst..

[8] Graham IM, Harris RN, Matejusová I, Middlemas SJ (2011). Do ‘rogue’ seals exist? Implications for seal conservation in the UK. Anim. Conserv..

[9] Linnell JDC, Aanes R, Swenson JE, Odden J, Smith ME (1999). Large carnivores that kill livestock: Do ‘problem individuals’ really exist?. Wildl. Soc. Bull..

[10] Tidwell, K. S., van der Leeuw, B. K., Magill, L. N., Carrothers, B. A. & Wertheimer, R. H. *Evaluation of pinniped predation on adult salmonids and other fish in the Bonneville Dam tailrace* (2017).

[11] Guillemette M, Brousseau P (2001). Does culling predatory gulls enhance the productivity of breeding common terns?. J. Appl. Ecol..

[12] Rudolf VHW, Rasmussen NL (2013). Population structure determines functional differences among species and ecosystem processes. Nat. Commun..

[13] Harmon LJ (2009). Evolutionary diversification in stickleback affects ecosystem functioning. Nature.

[14] Adams J (2016). A century of Chinook salmon consumption by marine mammal predators in the Northeast Pacific Ocean. Ecol. Inform..

[15] Chasco B (2017). Competing tradeoffs between increasing marine mammal predation and fisheries harvest of Chinook salmon. Sci. Rep..

[16] Bearhop S (2006). Stable isotopes indicate sex-specific and long-term individual foraging specialisation in diving seabirds. Mar. Ecol. Prog. Ser..

[17] Thiemann GW, Iverson SJ, Stirling I, Obbard ME (2011). Individual patterns of prey selection and dietary specialization in an Arctic marine carnivore. Oikos.

[18] Königson S, Fjälling A, Berglind M, Lunneryd SG (2013). Male gray seals specialize in raiding salmon traps. Fish. Res..

[19] Sih A, Sinn DL, Patricelli GL (2019). On the importance of individual differences in behavioural skill. Anim. Behav..

[20] Bjorkland RH (2015). Stable isotope mixing models elucidate sex and size effects on the diet of a generalist marine predator. Mar. Ecol. Prog. Ser..

[21] Schwarz D (2018). Large-scale molecular diet analysis in a generalist marine mammal reveals male preference for prey of conservation concern. Ecol. Evol..

[22] Tinker MT, Costa DP, Estes JA, Wieringa N (2007). Individual dietary specialization and dive behaviour in the California sea otter: Using archival time-depth data to detect alternative foraging strategies. Deep. Res. Part II Top. Stud. Oceanogr..

[23] Voelker MR, Schwarz D, Thomas A, Nelson BW, Acevedo-Gutiérrez A (2020). Large-scale molecular barcoding of prey DNA reveals predictors of intrapopulation feeding diversity in a marine predator. Ecol. Evol..

[24] Bolnick DI (2003). The ecology of individuals: Incidence and implications of individual specialization. Am. Nat..

[25] Harcourt R (1993). Individual variation in predation on fur seals by southern sea lions (*Otaria byronia*) in Peru. Can. J. Zool..

[26] Marine Mammal Commission (2004). Marine Mammal Protection Act. Marine Mammal Protection Act Amendment.

[27] National Marine Fisheries Service (2018). Willamette Falls Pinniped-Fishery Interaction Task Force Marine Mammal Protection Act, Section 120.

[28] Jefferson TA, Smultea MA, Ward EJ, Berejikian B (2021). Estimating the stock size of harbor seals (*Phoca vitulina richardii*) in the inland waters of Washington State using line-transect methods. PLoS ONE.

[29] Jeffries S, Huber H, Calambokidis J, Laake J (2003). Trends and status of harbor seals in Washington State: 1978–1999. J. Wildl. Manag..

[30] Thomas AC, Lance MM, Jeffries SJ, Miner BG, Acevedo-Gutiérrez A (2011). Harbor seal foraging response to a seasonal resource pulse, spawning Pacific herring. Mar. Ecol. Prog. Ser..

[31] Chasco B (2017). Estimates of chinook salmon consumption in Washington State inland waters by four marine mammal predators from 1970 to 2015. Can. J. Fish. Aquat. Sci..

[32] Farrer J, Acevedo-Gutiérrez A (2010). Use of haul-out sites by harbor seals (*Phoca vitulina*) in Bellingham: Implications for future development. Northwest. Nat..

[33] Steingass, S., Jeffries, S., Hatch, D. & Dupont, J. *Field report: 2020 pinniped research and management activities at Bonneville Dam* (2020).

[34] Tidwell KS, Carrothers BA, Blumstein DT, Schakner ZA (2021). Steller sea lion (*Eumetopias jubatus*) response to non-lethal hazing at Bonneville Dam. Front. Conserv. Sci..

[35] Hiruki LM, Schwartz MK, Boveng PL (1999). Hunting and social behaviour of leopard seals (*Hydrurga leptonyx*) at Seal Island, South Shetland Islands, Antarctica. J. Zool..

[36] Ainley DG, Ballard G, Karl BJ, Dugger KM (2005). Leopard seal predation rates at penguin colonies of different size. Antarct. Sci..

[37] Páez-Rosas D (2020). Hunting and cooperative foraging behavior of Galapagos sea lion: An attack to large pelagics. Mar. Mammal Sci..

[38] Macneale KH, Kiffney PM, Scholz NL (2010). Pesticides, aquatic food webs, and the conservation of Pacific salmon. Front. Ecol. Environ..

[39] Roni P, Anders PJ, Beechie TJ, Kaplowe DJ (2018). Review of tools for identifying, planning, and implementing habitat restoration for Pacific salmon and steelhead. North Am. J. Fish. Manag..

[40] Morissette L, Christensen V, Pauly D (2012). Marine mammal impacts in exploited ecosystems: Would large scale culling benefit fisheries?. PLoS ONE.

[41] Thompson D, Coram AJ, Harris RN, Sparling CE (2021). Review of non-lethal seal control options to limit seal predation on salmonids in rivers and at finfish farms. Scott. Mar. Freshw. Sci..

[42] Dickinson JL, Zuckerberg B, Bonter DN (2010). Citizen science as an ecological research tool: Challenges and benefits. Annu. Rev. Ecol. Evol. Syst..

[43] Fairbanks, C. & Penttila, D. *Bellingham Bay Forage Fish Spawning Assessment* (2016).

[44] Madsen SW, Nightengale T (2009). Whatcom Creek Ten-Years After: Summary Report.

[45] Martin P, Bateson P (2007). Measuring Behaviour: An Introductory Guide.

[46] Bolger DT, Morrison TA, Vance B, Lee D, Farid H (2012). A computer-assisted system for photographic mark-recapture analysis. Methods Ecol. Evol..

[47] Harrison PJ (2006). Incorporating movement into models of grey seal population dynamics. J. Anim. Ecol..

[48] Thompson PM, Wheeler H (2008). Photo-ID-based estimates of reproductive patterns in female harbor seals. Mar. Mammal Sci..

[49] Washington Department of Fish and Wildlife (2019). Whatcom Creek Hatchery.

[50] R Core Team (2020). R: A language and environment for statistical computing.

[51] Bates D, Mächler M, Bolker B, Walker S (2015). Fitting linear mixed-effects models using lme4. J. Stat. Softw..

[52] Lloyd-Smith JO (2007). Maximum likelihood estimation of the negative binomial dispersion parameter for highly overdispersed data, with applications to infectious diseases. PLoS ONE.

[53] Zhang, D. rsq: R-Squared and Related Measures. R package version 2.1 (2020).

[54] Lüdecke D, Ben-Shachar M, Patil I, Waggoner P, Makowski D (2021). Performance: An R package for assessment, comparison and testing of statistical models. J. Open Source Softw..

[55] Bolker BM (2009). Generalized linear mixed models: A practical guide for ecology and evolution. Trends Ecol. Evol..

[56] Zuur AF, Ieno EN, Walker N, Saveliev AA, Smith GM (2009). Mixed Effects Models and Extensions in Ecology with R.

