# Supplementary material for: Individual variability in foraging success of a marine predator informs predator management
Source: Sci Rep. 2022 Jul 1;12:11184. doi: 10.1038/s41598-022-15200-y (PMC9249773; doi:10.1038/s41598-022-15200-y)
Supplement: Supplementary file 1 — Supplementary Figures. [file 41598_2022_15200_MOESM1_ESM.docx]

**Full Title:** Individual Variability in Foraging Success of a Marine Predator Informs Predator Management

# Authors/affiliations:

*Grace Freeman: Western Washington University, [graceafreem@gmail.com](mailto:graceafreem@gmail.com)

Erin Matthews: Western Washington University, Skagit Fisheries Enhancement Group, [hello.erin.matthews@gmail.com](mailto:hello.erin.matthews@gmail.com)

Erin Stehr: Western Washington University, [erinstehr@gmail.com](mailto:erinstehr@gmail.com)

Alejandro Acevedo-Gutiérrez: Western Washington University, [aceveda@wwu.edu](mailto:aceveda@wwu.edu)

# Current Contact Information:

Grace Freeman

WI Dept. of Natural Resources Office of Applied Science 2801 Progress Rd

Madison, WI 53716 [graceafreem@gmail.com](mailto:graceafreem@gmail.com) (612) 708-9354


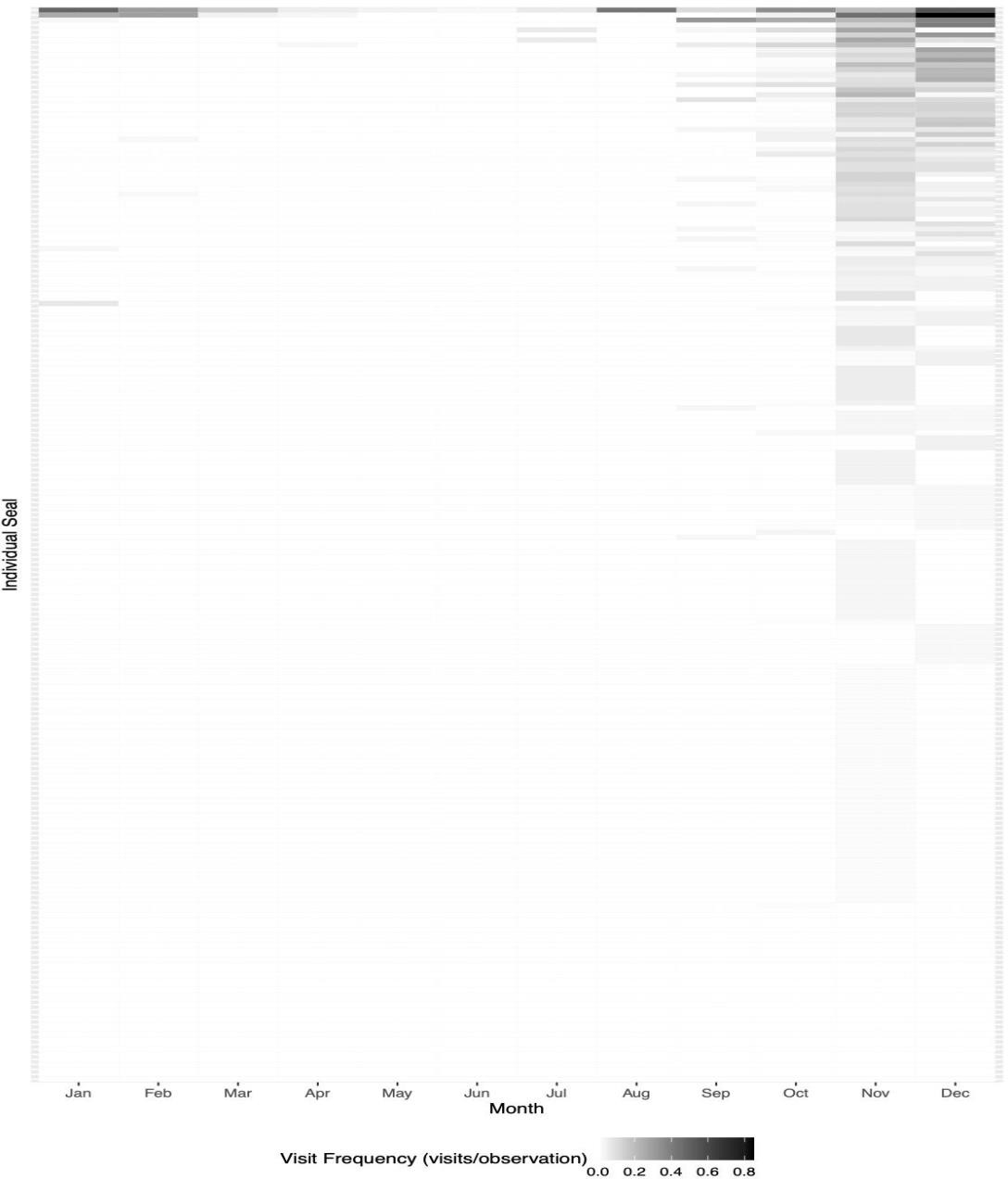


**Supplemental Figure 1**: Frequency of visit (number of visits per observation) by each individual harbor seal (n=170 seals) increased during run months (October - December). Each row corresponds to a single seal. Darker colors correspond to individuals with a higher number of visits per observation in a given month. Visit frequency was calculated by summing the total number of visits recorded by an individual during a given month across the study years and dividing by the total number of observation or sample days (i.e. total October visits/total October observation periods).


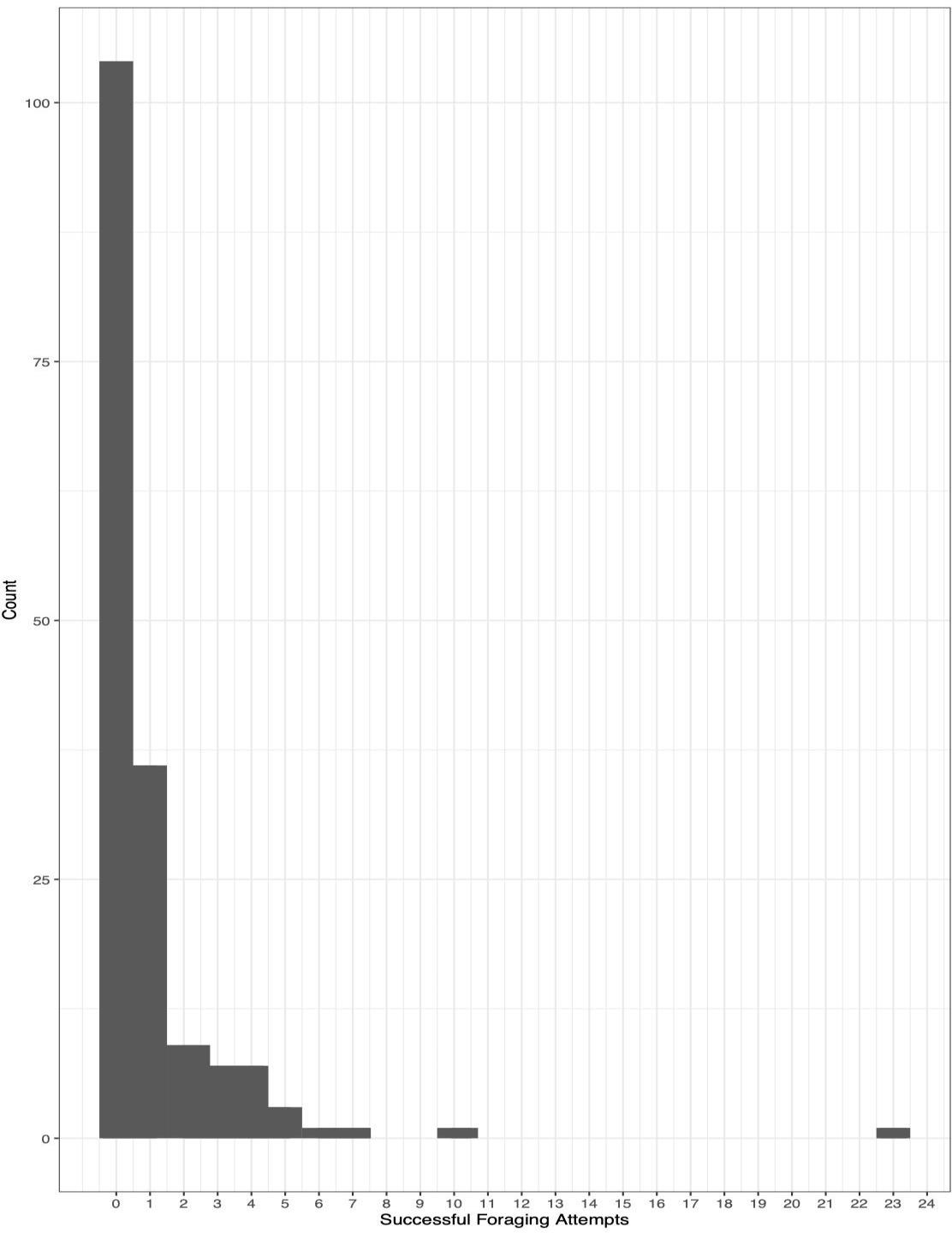


**Supplemental Figure 2:** Frequency histogram of successful foraging attempts for each individual harbor seal.
